# Some Therapeutical Indications for the Use of Jaborandi

**Published:** 1885-07

**Authors:** Q. C. Smith

**Affiliations:** Austin, Texas


### SOME THERAPEUTICAL INDICATIONS FOR THE USE OF JABORANDI.

*By Q. C. Smith, M. D., Austin, Texas.*

For Daniel's Medical Journal.

Stille tells us that, "the utility of jaborandi in medicine is confined to its power of lessening the amount of liquid in the system." See National Dispensatory, page 1062.

Prof. Stille's verdict, just quoted, is a striking illustration of the intense perniciousness that attaches to the impeding oracular dogmatic spirit that characterizes the writings of many learned and justly eminent men.

For chemical observation has demonstrated that jaborandi is not only a valuable remedy for "lessening the amount of fluid in the system," but is also a valuable remedy for many other curative purposes. To speak of jaborandi in general terms, we would say, that it is a remedy that will produce good results in most all cases in which it is desirable to diminish arterial blood-pressure, cool fever, or allay a sthenic hypersensative condition of the nervous system.

And it not only finds a legitimate field in the treatment of plethoric sthenic patients, who may be suffering from acute inflammatory diseases, but also in many cases where the patient, from prolonged ailment, has become greatly reduced in flesh and strength.

For instance, take a case of asthma of long standing, in which the usual line of routine orthodox and heterodox remedies have been plied in vain, the patient, as a result of prolonged, laborious breathing, is worn down and exhausted to an alarming degree, and every hour seems will be his last. Now begin the use of jaborandi by administering a hypodermic composed thus:

| | | |
|---|---|---|
| ℞ | Mur. Pilocarpine, | gr. 1-6, |
| | Apomorphia, | gr. 1-12. |

Mix and make a solution, and administer hypodermically. In many cases, within a few minutes, the patient is enabled to lie down and sleep—a precious boon he would not exchange for worlds of gold. This hypodermic dose will rarely need repetition, if the treatment is followed up as we will indicate. To give permanency to the good work so well begun, promptly follow up the hypodermic with something like the following:

| | | |
|---|---|---|
| ℞ | Fl. Ex. Jaborandi, | oz. i, |
| | Fl. Ex. Grindelia Robusta, | oz. ss, |
| | Syr. Ipecac, | oz. ss, |
| | Apomorphia, | gr. i, |
| | Comp. Syr. Stillingia, qs. ft., | oz. iv. |
| | Fiat, Sol. | |

S. Teaspoonful three times a day, just after meals, to be continued for several months.

Again. In almost all cases of acute pulmonic inflammatory diseases, especially in their *earlier* stages—before pleuritic or other effusions have occurred—jaborandi is a remdey of wonderful remedial power. By lowering arterial tension, it relieves pain, cools fever, equalizes the disturbed circulation, and thereby diminishes pulmonary congestion, and enables the patient to breath full, easy and naturally, and he drops into a gentle slumber, and as the observing old Coan Clinicien would say "the critical sweat comes on, the secretions, excretions and fluids of the body are properly concocted," and the convalescing patient—with proper after-treatment—rapidly recovers from a condition that our fathers would have considered hopeless without the vigorous use of the lancet, antimonials and mercurials, if perchance they should save the patient from the grave, to become a sad rebuke to the pernicious doctrines of Broussais and his blood-thirsty disciples.

Again. In the early congestive febrile stage of small pox, measles and scarlet fever, jaborandi—in small doses—is invaluable, for by its power to equalize the circulation, tranquilize the nervous system, it opens the depurative flood gates, internal and external, thereby enabling other appropriate remedies to produce their characteristic effects.

To speak in general terms, there are two important points concerning the therapeutic indications for the use of jaborandi, that are neglected or overlooked by many practitioners : It should be administered *early* in any given case, and in comparatively small doses. The book-doses are *too large* for general use, and its use usually deferred to too late a period or stage of the case. For if given early, before fluids accumulate in the cavities of the body, that grave complication will, in many cases, be averted.

Again. In the early stages of the so-called "irritative fevers" of small children, especially if there are convulsions, or even slighter nervous disturbance, jaborandi—which, here, may be combined with small doses of tincture gelsemium and bromide sodium—is an invaluable remedy.

Of course this—like other powerful remedies—is very liable to abuse, and should always be used carefully and with a clear understanding of the conditions to be remedied, and the peculiar power and characteristic action of the remedy. The immensely greater value that attaches to *prevention* above that of *cure*, would place jaborandi very high in the list of therapeutic agents. We have purposely left unsaid most of what standard works state in regard to the therapeutic indications, action and uses of jaborandi, deeming such repetition unnecessary. However, we do not presume, by any means, to have exhausted the subject.

